# Current status on health sciences research productivity pertaining to Angola up to 2014

**DOI:** 10.1186/s12961-015-0021-z

**Published:** 2015-07-01

**Authors:** Maria do Rosário Sambo, Albano V. L. Ferreira

**Affiliations:** Faculty of Medicine of Universidade Katyavala Bwila, Rua Sociedade de Geografia, Benguela, Angola

**Keywords:** Angola, Health research, Health research centres, Universities

## Abstract

**Background:**

Health research driven by the healthcare demands of the population can provide an informative evidence base to support decision-making processes on health policies, programmes, and practices. This paper surveyed the production of scientific research concerning health in Angola, specifically to access the publication rate over time, the main research topics and scientific fields, and the contribution of Angolan researchers and institutions.

**Methods:**

The study focused on data collected in a retrospective literature search in *Biblioteca Virtual em Saúde* (BVS) as of June 8, 2014, with the keyword “Angola” and on content information in correspondent publications deposited in PubMed.

**Results:**

BVS generated 1,029 hits, 74.6 % of which were deposited in PubMed where 301 abstracts were described. From 1979 to 2003, there were 62 publications and in 2004–2013 the quantity increased four-fold (*n* = 232); malaria was the most frequent topic (*n* = 42). Angola was the country with the largest number of publications, taking into account the primary affiliation of the first author (*n* = 45). Universities, institutes, or research centres accounted for 65 % of the publications and in descending order Portugal, Brazil, and the United States of America occupied the three first positions. Epidemiology was by far the most frequent field of research (*n* = 165).

**Conclusions:**

The number of publications has increased steadily over the past 10 years, with predominance on malaria topics. Angola was the country with the largest number of major affiliations of the first author, but the contribution of Angolan institutions was relatively low, indicating a need to reinforce academic research institutions in the country.

## Background

The concept of research for health, expressed on the World Health Report 2013, covers a broader range of investigations than health research. This wider view of research will become increasingly important in the transition from the United Nations Millennium Development Goals to a post-2015 sustainable development agenda [1].

In a more restricted concept, health research has the potential to contribute to the identification of social and economic determinants of health. In the context of implementation research, it is a major basis for decision-making processes on health policies, programmes, and practices, especially when driven by the demand of the health problems of the population [2, 3].

Moreover, conducting more health research can stimulate countries to strengthen their capacity to produce and use such research to guide decision-making, improve the efficiency of the investments, increase accountability in the work of researchers, and contribute substantially to the research training of human resources in developing countries [4, 5]. In the perspective of the World Health Organization (WHO), health research must be founded on building capacity to step up health research systems and support demand-driven health problems, particularly in low- and middle-income countries. Additionally, health research must be embedded with standards for best practices and ensure that quality evidence is turned into affordable health technologies and evidence-informed policy [6].

However, at the global level, the proportion of health research that considers these aspects is negligible [7]. In particular, evidence shows that the research output in developing countries is considerably less than in developed countries [8]. Therefore, it is crucial to get an accurate picture of the landscape of the scientific research produced in developing countries in order to identify gaps critical for social development [9]. In sub-Saharan Africa, in particular, the production of health research can affect teaching, the quality of undergraduate training, career promotion, and translational research relevant to the country, which is comparable to developed countries [5].

There have been documented bibliometric studies of research production in Africa, as a whole, as well as in other continents. This then motivated studies of the chronological progression of research within individual African countries [10–18]. These publications demonstrate the feebleness of African authorship in the most cited scientific publications in health [19].

Angola, a Portuguese-speaking country, is independent since 1975 and has spent 27 years in progressive socio-political instability, with the drawback of the civil war that ended 12 years ago. Nevertheless, Angola is making progress towards the achievement of the health Millennium Development Goals but is still far from achieving them [1]. The reconstruction of the national health system and policy will benefit from the knowledge of the factors influencing the success of certain interventions and the measure of their impact on populations. Therefore, health research will contribute to increased efficiency and effectiveness of health policy and decision making processes in Angola on programmes and practices. Support for science in Angola is less than 0.1 % of its GDP, compared to a world average of 1.7 % [20]. Consequently, a more serious commitment would be needed to build local and national capacity to accelerate the improvement of human resources training for health research. In this context, it becomes even more appropriate to carry out a survey on what has been produced as the result of health research. When searching the literature on health research in Angola, we found only one publication dated from 1953 which linked research to health, entitled “Scientific Research and the Health Status of Angola” [21]. The lack of studies on health research produced in Angola in the last 61 years motivated this work.

We carried out a non-systematic literature survey to evaluate the progression of health research pertaining to Angola, answering specifically the following questions: 1) how has the number of publications evolved over the years? 2) what are the most published topics? 3) what is the level of involvement of Angolan researchers and institutions in this published research? and 4) what are the most represented research fields?

We expect that this study will contribute to increasing the current state of knowledge on health research related to Angola and identify gaps that may guide the WHO recommendations on health research and consequently the adoption of effective interventions that will strengthen health research in Angola.

## Methods

We used the reference database *Biblioteca Virtual em Saúde* (BVS) to perform a non-systematic scientific literature survey aiming to count all existing publications up to June 8, 2014, with the keyword “Angola” and to produce a scientific publications dataset to be compared with other databases. The BVS was chosen because it is a platform that covers human health sciences issues, information sources published in countries of the Portuguese-speaking community, and a set of international databases. Moreover, resource constraints did not allow access to other biomedical databases such as those using the OVID platform. Thereafter, our content information study was based on the public database MEDLINE/PubMed disclosing the best match with the BVS dataset using the same keyword.

Publications with no abstract available were excluded from the MEDLINE/PubMed dataset. To focus our study in human health research relevant for Angola, we revised the abstracts using the following exclusion criteria: 1) the word Angola did not mean the country; 2) the topic did not concern physical and mental health; 3) the topic reported exclusively to biological vectors; 4) Angola was not the focus of a specific study, but was instead reported as part of a group of countries (common for poliomyelitis and Marburg disease) or just as a reference; 5) case reports published outside Angola regarding citizens who temporarily resided in Angola.

From each publication included in the study we extracted the following variables: year of publication; topic of health or disease, according to the revised International Classification of Diseases (ICD-10); country in which the primary institution of the first author affiliation was based; type of institution of the first author primary affiliation (university, institute or centre of scientific research, hospital, non-governmental organization, other); presence of an Angolan citizen as first author; number of Angolan co-authors; field of research (basic biomedical, clinical, epidemiological or socioeconomic and professional); and publishing journal. The Angolan authors and co-authors were identified by consulting their registration on the National Medical Council and in the universities. Socioeconomic and professional research includes health policy, human resources, and governance and management of health systems. The year of publication was the single variable that did not require the existence of the abstract. All the references of the abstracts included in the study were stored in a collection in the Zotero library. Collection of abstract content data was performed by manual curation and data were entered into an excel spreadsheet (Microsoft™ Excel® 2007) for statistical processing and plots generation.

## Results and discussion

Searching the BVS on June 8, 2014, with the keyword “Angola”, showed 1,029 results, of which 74.6 % were also in MEDLINE/PubMed database. Consequently, it was considered that further search in MEDLINE/PubMed would produce representative research publication data on health in Angola. Therefore, we undertook a further search for relevant Angolan publications in MEDLINE/PubMed, keeping the keyword “Angola”. To describe the number of publications across time we included all publications retrieved from BVS without the abstract filter. All other variables were taken into account using the availability of an abstract as the main inclusion criteria, and this yielded 658 abstracts. Applying the exclusion criteria we selected 301 abstracts for further description.

### Publication rate

**Fig. 1:**
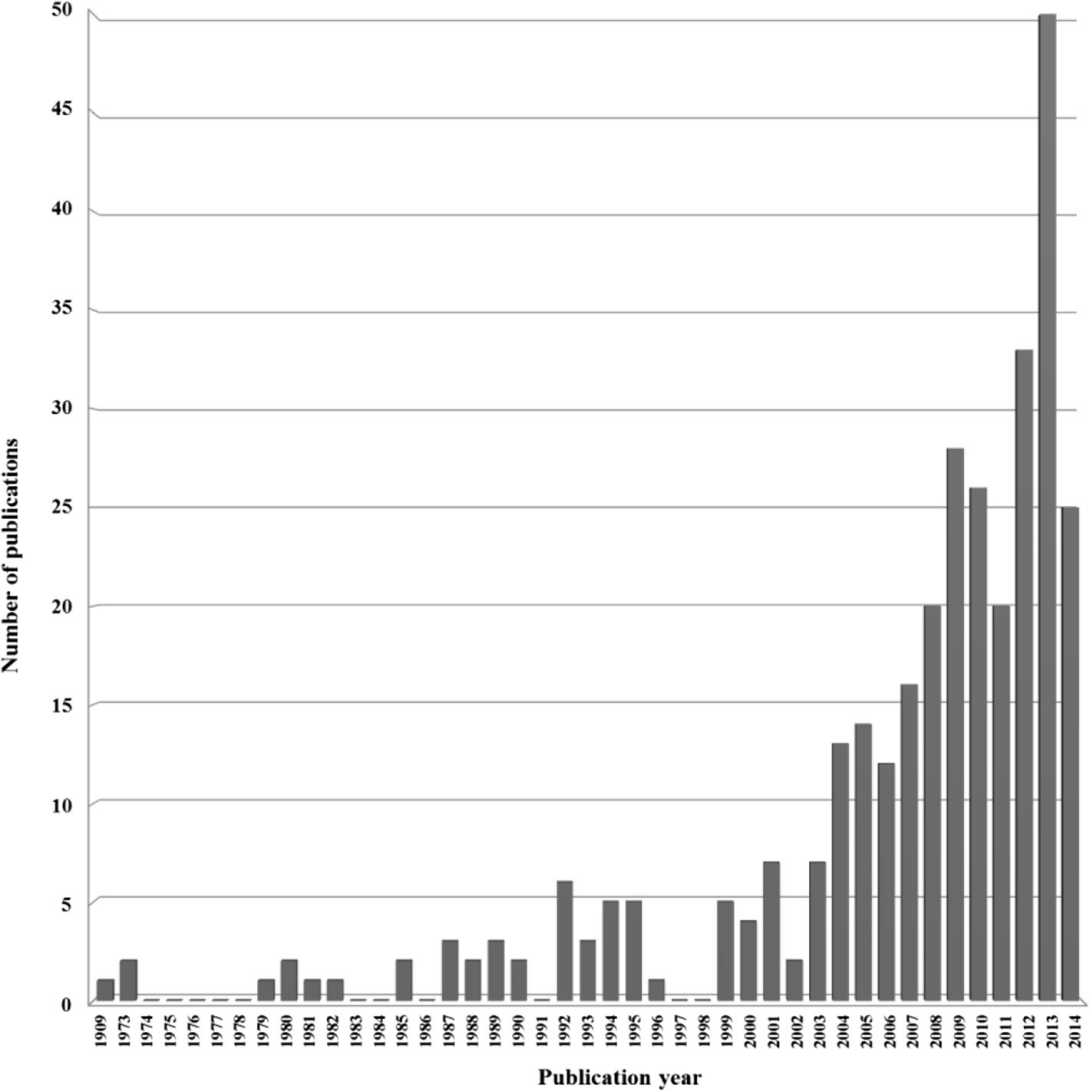
Between 1909 and 1973, none of the publications deposited in MEDLINE/PubMed up to June 8, 2014, complied with the inclusion criteria for abstracts about health research in Angola

As shown in Fig. 1, the publications in MEDLINE/PubMed addressing health issues related to Angola date back to 1909. MEDLINE/PubMed, the first bibliographic database in the life sciences, with a focus on biomedicine, mainly covers the literature published from 1946 onwards, but it also includes more dated publications [22]. There has been a remarkable, albeit irregular, increase in the number of publications during the 1990s, with a more sustained increase from 2004 onwards, and a peak in 2013. From 1979 to 2003 (34 years) there were 62 publications, but in the last 10 years (2004–2013) the number increased four-fold (n = 232), suggesting a rampant increment in publication rate (Fig. 1). Additionally, it is noteworthy that the number of publications by the beginning of June 2014 already reached half the figure recorded in 2013, supporting the notion that Angola is motivating a steady increase in health-related scientific publications. This exponential trend was also observed in Palestine, a war zone, where a total of 770 publications were retrieved in the medical and biomedical field across a 10-year period (01 January 2002 to 31 December 2011), averaging approximately 80 articles per year. Interestingly, the number of publications has also increased four-fold during the period 2002–2011, with a stabilization in the last 3 years of the study period [23].

A study published in 2007, which analyzed the geography of the PubMed biomedical publications in Africa between 1996 and 2005, concluded that the contribution of Africa, in particular, was considerably less than that of other continents. Indeed, it was evident that there was a continuous increase in publication output during this period in all African sub-regions, although Angola was located in the lowest quintile with less than two publications per year [17]. In the present study, we found, in the same period, five publications that included researchers that were affiliated with Angolan institutions. However, this did not change the lower position of Angola. Another publication about the scientific production in Public Health and Epidemiology in the WHO African region, in the period 1991–2010, obtained very similar results for Angola, placing it in the lowest quintile, with 11–50 publications [18]. This increase in health research in Angola matches with a recent study about the WHO African Region between 2000 and 2014 [24]. In this study, Angola was located in quintile three, with 100 to 499 articles.

### Health research topics

**Fig. 2:**
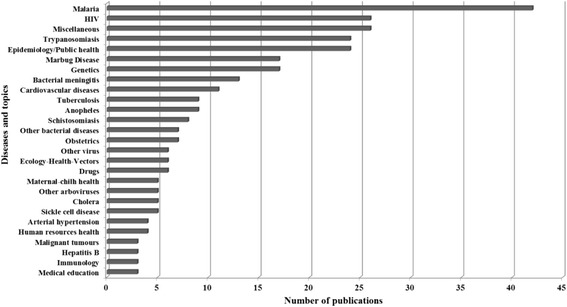
Publications are deposited in MEDLINE/PubMed up to June 8, 2014

Malaria was the disease that stood out, with 45 publications, followed by HIV infection (*n* = 26), trypanosomiasis (*n* = 24), and themes of epidemiology/public health (*n* = 24). Tuberculosis occupied the tenth position with nine papers (Fig. 2). As a whole, infectious diseases was a topic of 59 % of the publications under analysis (*n* = 178).

The most investigated topics are aligned with the epidemiological profile of Angola, regarding the main causes of morbidity and mortality (communicable diseases), with a large emphasis on malaria [25]. Interestingly, there was a disproportionate volume of publications on HIV/AIDS relative to tuberculosis, which is unusual given the frequent association of both morbidities [26]. In an assessment of scientific output in public health and epidemiology in Africa, in the period 1991–2010, HIV/AIDS infection was the predominant topic (11.3 %) while malaria accounted for 8.6 % and tuberculosis for 7.1 % [16]. This analysis also considers the issue of the importance of research funding concerning infectious diseases [27]. The trend towards epidemiological transition in sub-Saharan Africa, owing to the increase of chronic non-communicable diseases, is not yet reflected at the scientific publication level, neither in the present study nor in the aforementioned African publications [16, 28–30].

### Authorship and affiliation

**Fig. 3:**
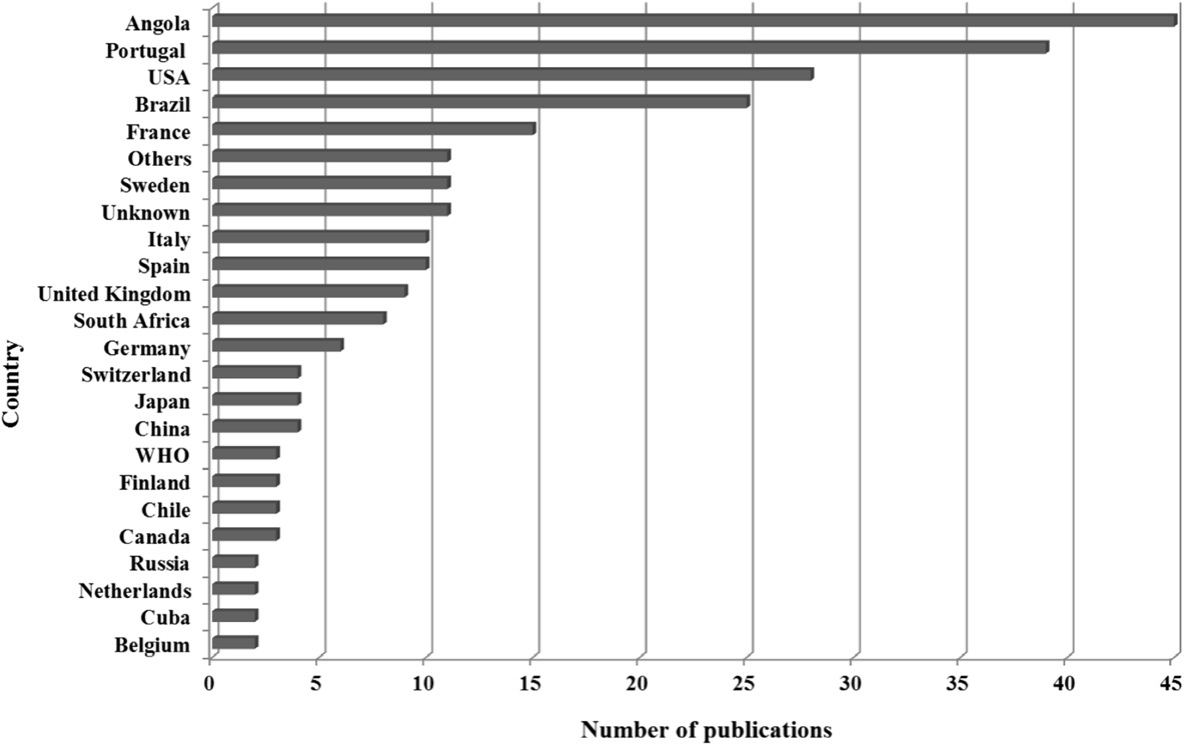
Country denotes the location in which the primary institution of the first author affiliation was based in publications up to June 8, 2014

**Fig. 4:**
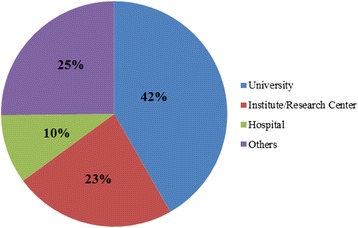
Publications deposited in MEDLINE/PubMed up to June 8, 2014

**Fig. 5:**
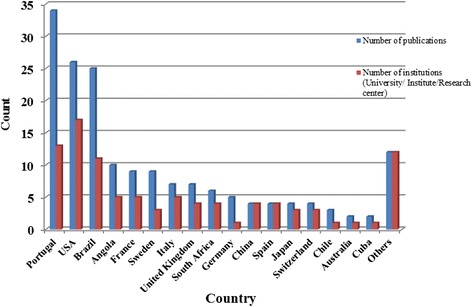
Country denotes the location in which the primary institution of the first author affiliation was based concerning publications on health research in Angola deposited in MEDLINE/PubMed up to June 8, 2014

The primary affiliation of the first author was most frequently in an Angolan institution (45 publications) (Fig. 3). A total of 150 institutions were listed as the first author’s primary affiliation, revealing a high institutional dispersion; 14.7 % were Angolan institutions. Furthermore, 65 % of publications represented research conducted in universities and institutes or research centres (Fig. 4). The majority of publications by first author affiliation country and the number of academic research institutions were located in Portugal, United States of America, or Brazil (Fig. 5). The high number of publications with first author affiliation in Angola denotes the concern of researchers in linking their work to institutions from the country of study. Nevertheless, this involvement is dispersed across many different institutions, many of which have relatively low track records in publications, namely hospitals and non-governmental organizations. Furthermore, academic research in universities and institutes (or research centres) related to institutional affiliations abroad indicate the need for reinforcing the academic research capacity in Angola.

### Publications by Angolan researchers

**Fig. 6:**
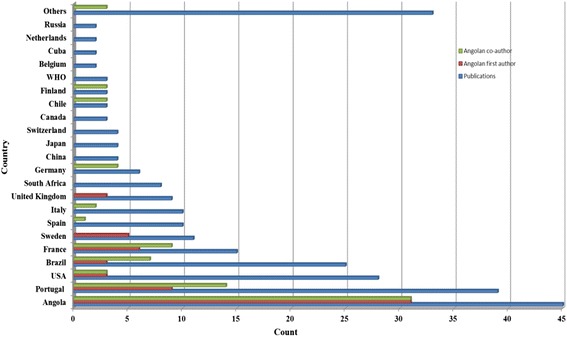
Publications on health research in Angola deposited in MEDLINE/PubMed up to June 8, 2014, according to country in which the primary institution of the first author affiliation was based

An Angolan researcher was the first author in 58 (19 %) abstracts. The frequency of Angolan first authors or co-authors increased when the first author was affiliated to an Angolan institution (Fig. 6). In the present study, it is noteworthy that one-fifth of all publications had an Angolan as first author. The number of African first authors on publications relating to Africa between 1991 and 2010 has been increasing [16]. This finding was reinforced in the period between 2000 and 2014 in the WHO African Region, and it was found that the contributions of first authors from Africa doubled in this period, although it is recognized that it is still minimal (0.7 % in 2000 and 1.3 % in 2014) [24].

### Scientific fields

**Fig. 7:**
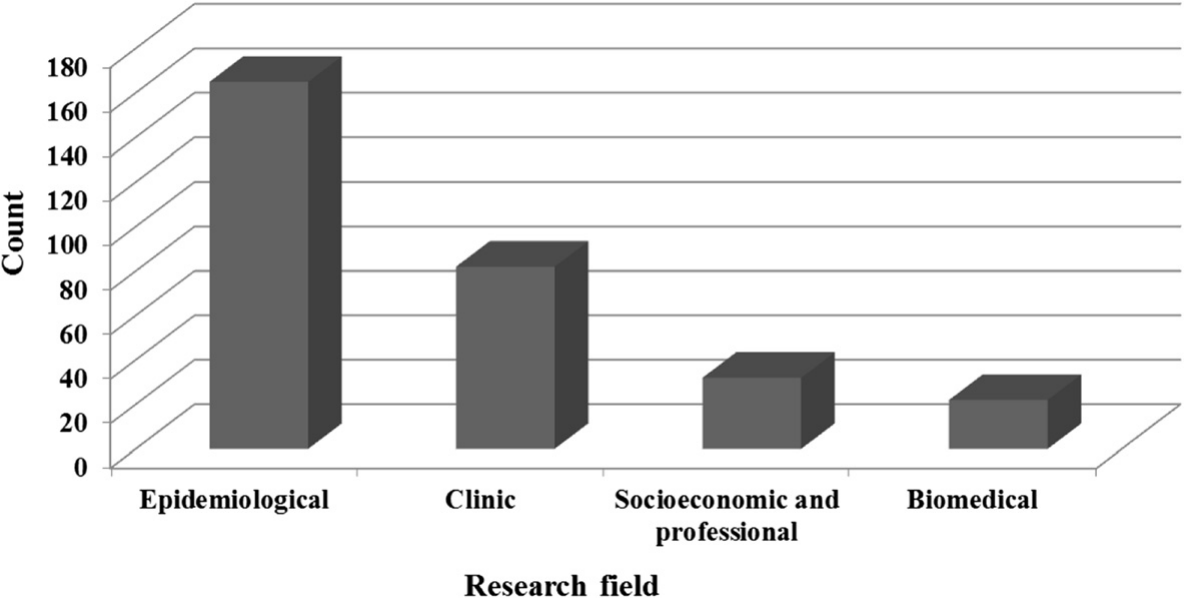
Publications deposited in MEDLINE/PubMed up to June 8, 2014

Epidemiological research was by far the most common research area (*n* = 165) (Fig. 7). Although a few population genetics studies (data not shown) are included in this topic, this finding is in accordance to the burden of infectious diseases in the country. However, the increasing frequency of non-communicable diseases in sub-Saharan Africa is likely to be a matter of concern for research in Africa in coming years.

On the other hand, a small number of publications (*n* = 32; 10.6 %) on socioeconomic and professional research, including governance, health policies, health systems management, and human resources is noted. Considering that Angola was a war zone for more than 50 % of its existence as an independent state, we might have expected a larger number of research publications on relevant topics such as the socioeconomic determinants of health (e.g., unemployment, poverty, education, family dissolution, and lifestyles) and the performance of health unities and health systems. However, we recognize the possibility that studies concerning some of those socioeconomic determinants of health may be part of research in social and economic sciences and, therefore, more accessible in other bibliographic databases. Further, the lack of studies on human resources in the various health professions, including studies in medical education, is also noticeable.

We expect that the increasing number of health professional schools in Angola, including six new public medical schools, particularly since 2009, will raise interest in these topics, especially if educational curricula at the undergraduate and postgraduate levels in Angola incorporate more programs focused on improving research skills and providing on-the-job training in epidemiology [31].

### Journals choice

**Fig. 8:**
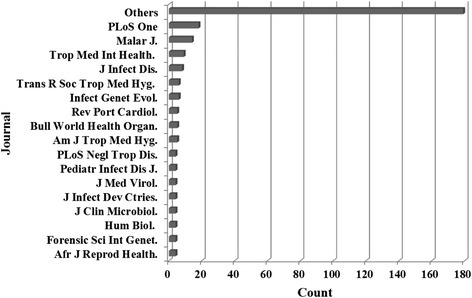
Publications on health research in Angola deposited in MEDLINE/PubMed up to June 8, 2014

We also found that, despite publications being widely spread in a variety of journals, 11.4 % concentrated in *PLoS ONE* and *Malaria Journal*, both of which are free access journals indexed by common databases (Fig. 8). This finding is not unusual given that malaria was the most common research topic among Angolan publications observed in the present study. Notwithstanding the debate around open access publishing, our findings reinforce the importance of providing readers in financially disadvantaged countries with a wide range of relevant research and information [32, 33].

### Limitations of the study

The fact that Angola is a Portuguese speaking country and the majority of the journals indexed by MEDLINE/PubMed are in English may have introduced a selection bias due to idiom barriers. Though MEDLINE/PubMed represented the vast majority of the publications of the study and the search began at BVS, which included papers published in journals indexed by Scielo, it does not embody all the scientific and biomedical journals. Furthermore, we did not search “grey literature”. Thus, the number of Angolan authors and coauthors might be underestimated. Nevertheless, Angola still does not have an exhaustive electronic database of researchers containing individual publication records. In addition, the incorrect reporting of authors’ nationality and affiliation, observed in a few papers in the present study, may further contribute to underestimation of Angolan-authored publications. Finally, the study did not include the analysis of journal impact factors and the authors did not have access to any citation index database, which would allow the performance of bibliometric citation analyses.

## Conclusions

This initial contribution characterizing the productivity of health research pertaining to Angola showed that the number of publications has increased steadily over the past 10 years and was mainly focused on infectious diseases, namely malaria. Angola, as the country in which the primary institution of the first author affiliation was based, evidenced the largest number of publications, and about 20 % of the publications included an Angolan as the first author. However, academic institutions in Portugal, the United States of America, and Brazil contributed more than universities and research centres in Angola to research publication. The research was largely epidemiological, and remarkably, with a very small number of publications on economic and professional issues, involving themes such as health policy, governance, and management of health systems and human resources.

In Angola, with the large expansion of higher education since 2009, universities should build and strengthen their capacities in research and human resources training. Including research training in the curricula of pre-graduate courses in health sciences will contribute to graduate professionals able to act as agents responsible for change, competent human resource managers, and promoters of evidence-based policies [34]. The alignment of the national health research agenda with the national program of health development and the wide use of multilateral international cooperation are critical to develop the operational tools towards universal health coverage in Angola as conceived by the WHO [1]. In summary, this work highlights the rapid increase of scientific publications related to Angola but also a need for reinforcing academic-driven research in this country.
